# Changes of Flavan-3-ols with Different Degrees of Polymerization in Seeds of ‘Shiraz’, ‘Cabernet Sauvignon’ and ‘Marselan’ Grapes after Veraison

**DOI:** 10.3390/molecules15117763

**Published:** 2010-11-02

**Authors:** Yan-Xia Liu, Qiu-Hong Pan, Guo-Liang Yan, Jian-Jun He, Chang-Qing Duan

**Affiliations:** Centre for Viticulture and Enology, College of Food Science & Nutritional Engineering, China Agricultural University, Beijing 100083, China; E-Mails: yanxia_liu@foxmail.com (Y.-X.L.); panqh@cau.edu.cn (Q.-H.P.); yangl@vip.sohu.com (G.-L.Y.); hejj.email@gmail.com (J.-J.H.)

**Keywords:** flavan-3-ols, grape seeds, degree of polymerization, normal-phase HPLC-MS

## Abstract

Flavan-3-ols consist of flavan-3-ol monomers and polymers with different degrees of polymerization (DP). In this study, flavan-3-ol extracts from grape seeds were well separated into three fractions including monomers, oligomers (2 < DP < 10) and polymers (DP > 10), by means of normal-phase HPLC-MS. The different patterns of these three fractions were analyzed in three *Vitis vinifera* cultivars (‘Shiraz’, ‘Cabernet Sauvignon’ and ‘Marselan’) seeds from veraison to harvest. The results showed: (1) polymers were the main form of flavan-3-ols in grape seeds and monomers accounted for only a small proportion; (2) the contents of flavan-3-ol monomers in the seeds of three grape cultivars all exhibited a gradually decreasing trend with a little fluctuation, whereas the patterns of the change of contents of oligomers and polymers were extremely different among grape cultivars; the contents of flavan-3-ol oligomers were enhanced in the seeds of ‘Cabernet Sauvignon’, but were reduced in the other two cultivars; (3) with regard to the proportion of flavan-3-ols with a certain DP to total flavan-3-ols, both flavan-3-ol monomers and flavan-3-ols with low DP fell in proportion, while the flavan-3-ols with high DP increased correspondingly. These findings indicate that flavan-3-ol polymerization in developing seeds is variety-dependent and may be genetically regulated.

## 1. Introduction

Flavan-3-ols are widely distributed in higher plants like grape. Abundant flavan-3-ols are present in grape skins and seeds [1,2,3], and are transferred into wine during must fermentation [4,5], providing wine with organoleptic properties such as astringency, bitterness, and colour stability [6,7]. 

The exact or mean degree of polymerization of flavan-3-ols is a major factor influencing the sensory properties of bitterness and astringency [8,9,10,11,12]. Flavan-3-ols are generally divided into three groups according to their DP: monomers, oligomers and polymers. The oligomeric and polymeric flavan-3-ols are also called proanthocyanidins or tannins more often by enologists. Favan-3-ol monomers have been found to be more bitter than oligomers [8]. The relatively low-polymerized flavan-3-ols (monomers, dimers, trimers, *etc*.) taste more acid than astringent [9]. Oligomeric and polymeric procyanidins give wine obvious bitterness and astringency [10]. According to the report of Peleg *et al.* [8], the astringency of proanthocyanidins, at equal concentrations, increases with chain length up to the decamer level, but declines when the proanthocyanidins are larger than decamers, because the polymers become insoluble. However, this view has been subsequently questioned. Maury *et al.* [11] have determined the existence of proanthocyanidins with higher DP (>20) in red wine; and Vidal *et al.* [12] also have found that astringency becomes more intense as mean degree of polymerization (mDP) increases, but chain length has no effect on bitterness perception, irrespective of proanthocyanidin classes. As a result, flavan-3-ol polymerization characterizes, to some extent, grape and wine flavor.

With the development of chromatographic and spectroscopic methods, quantization of individual small molecular size phenolic compounds in grapes has become possible [13], and some effective separation methods for polymeric flavan-3-ols according to their DP have been established [14]. For example, gel chromatography with different gels such as Sephadex LH-20, TSK HW-40, or C_18_ Sep-Pak cartridges has been used [15,16,17]. In addition, based on relative solubility of flavan-3-ols in different solvents (methanol and chloroform), separation may be achieved by dissolution procedures or successive precipitations [18,19]. However, these methods are time and reagent consuming. Normal phase HPLC can separate flavan-3-ols with different molecular sizes rapidly with production of less waste [20], and the resolution of these compounds has been improved recently [21]. Usually, the DP of these compounds may be determined by reverse-phase HPLC analysis after thioacidolysis [22,23,24]. By this method, only mDP, but no information about the molecular mass distribution can be obtained. 

Considerable research has been done to describe the flavan-3-ol changes during grape berry ripening [2,25,26,27,28,29]. Similar trends of flavan-3-ols during berry development have been reported, that is, the generation of flavan-3-ols in grape berries commences with the flowering stage and flavan-3-ols accumulate rapidly towards a maximum around veraison, followed by a decrease at subsequent ripening stage. In these studies, flavan-3-ols with low DP (DP ≤ 4) were mainly measured using reverse-phase HPLC, and polymeric flavan-3-ols were analyzed mostly through acid-catalyzed cleavage in the presence of excess phloroglucinol or benzyl mercaptan [22,23,24]. These researches mostly concern the contents of monomeric flavan-3-ols and mDP of the polymeric flavan-3-ols, rather than the contents of various oligomeric or polymeric flavan-3-ols. Up to now, no detailed information has been given on change patterns of oligomeric or polymeric flavan-3-ols with a certain DP during berry maturation. 

Although the biosynthesis of flavan-3-ol units is well known, the mechanism of flavan-3-ol polymerization remains unclear. Grape seeds are rich in flavan-3-ols, and thus can be used as an ideal material to study this polymerization during berry maturation. In this study, we fractioned flavan-3-ol extracts from grape seeds into three fractions according to their DP by normal phase HPLC-MS, and quantified each fraction. On this basis, the changes of flavan-3-ols with different DP were investigated in ripening seeds of three winegrape cultivars (‘Shiraz’, ‘Cabernet Sauvignon’ and ‘Marselan’) after veraison. This work will provide some valuable information for ultimately uncovering mechanism of flavan-3-ol polymerization. 

## 2. Results and Discussion

**Figure 1:**
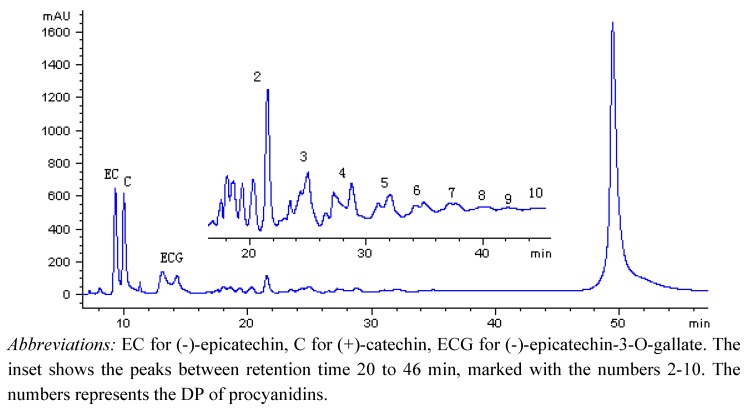
NP-HPLC chromatogram of a ‘Shiraz’ grape seed extract monitored at 280 nm.

**Figure 2:**
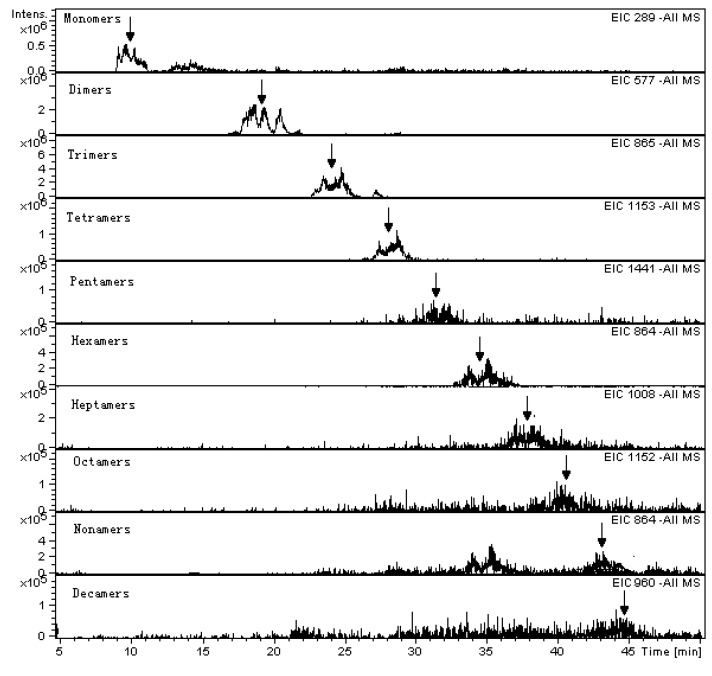
Extracted ion chromatogram of non-galloylated flavan-3-ol (DP = 1～10) from the total ion chromatogram on negative ESI-MS. The peaks are indicated by arrows.

Flavan-3-ol monomers to decamers were well separated and the polymers larger than decamers were eluted as a single peak corresponding to a retention time of 50 min (Figure 1). The monomer peaks were identified as (-)-epicatechin (EC), (+)-catechin (C), and (-)-epicatechin-3-O-gallate ECG), respectively, according to their mass spectra and comparison with the retention time of the external standards (Table 1). The oligomers were characterized as dimers to decamers in the light of their Extracted Ion Chromatograms (Figure 2) from the Total Ion Chromatogram. Based on this method, individual monomeric flavan-3-ols could be exactly measured in quantity, while the oligomeric and polymeric falvan-3-ols could be relatively quantified by using the standard procyanidin B1 as reference.

**Table 1:** HPLC-UV-MS/MS spectral information of flavan-3-ol compounds identified in grape seeds.

Retention time (min) (min)		Degree of Polymerization	m/z of ions		
			[M-H]^-^	[M-2H]^2-^/2	[M-3H]^3-^/3
	9.9	1	289		
	20.0	2	577		
	25.0	3	865		
	29.0	4	1153	576	
	32.0	5	1441	720	
	34.0	6	1729	864	
	38.1	7		1008	672
	41.2	8		1152	768
	43.0	9		1296	864
	45.0	10		1441	960

### 2.1. Changes in monomeric flavan-3-ols after veraison

**Figure 3:**
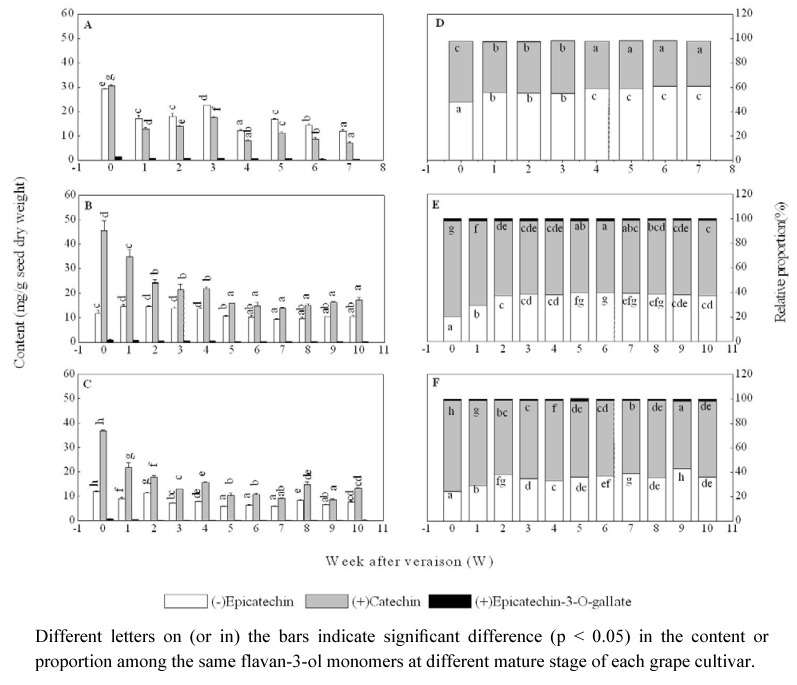
Changes in content and relative proportion of (-)-epicatechin, (+)-catechin and (‑)-epicatechin-3-o-gallate in ‘Shiraz’ (A, D), ‘Cabernet Sauvignon’ (B, E) and ‘Marselan’ (C, F) grape seeds after veraison.

Three flavan-3-ol monomers, C, EC, and ECG, were detected, but was ECG present in trace amounts, representing less than 1% of the total concentration of flavan-3-ol monomers. In the seeds of ‘Shiraz’ grape, EC was the major flavan-3-ol monomer during whole experiment, except for the first sampling date ( Figure 3A-C ), while (+)-catechin (C) was the most abundant flavan-3-ol monomer in ‘Cabernet Sauvignon’ and ‘Marselan’ seeds, which is consistant with previous reports [22,23]. For each grape cultivar, three monomers all showed a fluctuating decline in their contents from grape veraison to commercial harvest, and a rapid decrease in total content occurred from the onset of veraison to four weeks after veraison (Figure 3). It has been suggested that the decrease of flavan-3-ol content in grape seeds is probably due to flavan-3-ol polymerization and oxidation [30,31] or flavan-3-ols binding with other components, such as proteins and polysaccharides [22,23,26,31,32]. As a result of this, extractable flavan-3-ols decrease. 

Regarding the relative proportion of the contents of individual monomer to total content of all flavan-3-ol monomers at the same stage, EC increased significantly in all three of these cultivars with seed maturation, from 47.8% to 60.7% for ‘Shiraz’, from 20.4% to 37.3% for ‘Cabernet Sauvignon’ and from 24.2% to 35.9% for ‘Marselan’ (Figure 3), respectively. This increase in the relative proportion of EC is in agreement with the previous reports [22,23,31]. Meanwhile, the relative proportion of C decreased at almost the same rate, whereas that of ECG did not change significantly during the experimental period (Figure 3). This indicates that reduction of C content is more than that of EC during seed maturation. Interestingly, the relative proportion of EC content is always higher than that of C content in developing ‘Shiraz’ seeds, and just the opposite in the ‘Cabernet Sauvignon’ and ‘Marselan’ seeds.

### 2.2. Changes in oligomeric flavan-3-ols after veraison

**Figure 4:**
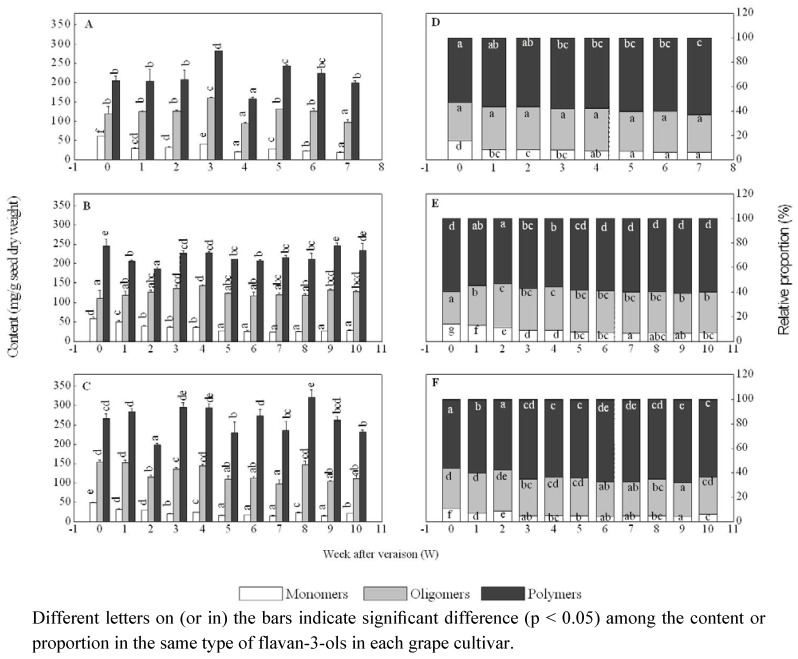
The content and relative proportions of monomers, oligomers and polymers in ‘Shiraz’ (A, D), ‘Cabernet Sauvignon’ (B, E) and ‘Marselan’ (C, F) grape seeds after veraison.

Total content of oligomers (2 < DP < 10) behaved differently in these three grape cultivars. It decreased with fluctuations in the seeds of both ‘Shiraz’ and ‘Marselan’, but an increase in total content of oligomers was observed in ‘Cabernet Sauvignon’ (**Figure 4**). At harvest, the seeds of ‘Cabernet Sauvignon’ contained the highest level of total flavan-3-ol oligomers (about 130 mg/g.DW). 

**Table 2:** Content of individual oligomers in ‘Shiraz’, ‘Cabernet Sauvignon’, ‘Marselan’ grape seeds after veraison.

Grape cultivar	Weeks after veraison	Content (mg/g seed DW)														
		P2		P3		P4		P5		P6		P7		P8-10		
		mean	SD	mean	SD	mean	SD	mean	SD	mean	SD	mean	SD	mean	SD	
	0	26.56b	3.43	14.15b	1.90	14.69b	2.02	11.16ab	2.01	10.24ab	2.12	9.97abc	2.58	33.29b	5.21	
	1	25.75b	0.77	14.73b	0.27	15.89bc	0.57	12.80ab	1.25	10.94bc	0.35	11.23bc	1.18	33.69b	1.45	
	2	26.15b	0.85	14.82b	0.23	16.14bc	0.64	13.42bc	1.03	11.19bc	0.38	10.75abc	0.35	34.21b	1.03	
‘Shiraz’	3	32.80c	0.17	19.27c	1.15	21.22d	0.13	17.36d	0.12	14.74d	0.25	13.93d	0.97	41.46c	1.29	
	4	18.21a	0.73	11.69a	0.19	12.32a	1.04	10.34a	0.82	8.93a	0.41	9.34ab	0.34	23.79a	2.19	
	5	25.11b	0.98	15.90b	0.41	17.64c	0.67	14.33c	0.91	12.22c	0.33	12.19cd	1.01	34.20b	3.56	
	6	23.58b	1.35	14.92b	1.12	16.08bc	0.49	13.60bc	0.95	11.71bc	0.96	11.28bc	0.48	35.39b	1.05	
	7	19.13a	1.27	11.74a	1.06	12.57a	0.71	10.56a	1.19	8.88a	0.76	8.80a	0.30	26.08a	1.91	
	0	22.71a	4.13	13.48a	2.65	14.30a	2.01	11.14a	2.15	10.49a	2.52	9.91a	1.82	27.91a	7.17	
	1	24.20a	1.28	13.81a	0.80	15.51ab	0.79	12.59a	1.58	11.79ab	1.77	11.15ab	2.22	30.49ab	3.94	
	2	25.22ab	0.53	15.90ab	0.34	16.63ab	0.47	13.28ab	0.76	12.03ab	1.67	11.98ab	1.28	31.57ab	1.95	
	3	25.21ab	1.78	16.56ab	1.46	17.61b	1.25	14.74ab	1.05	13.00ab	1.08	12.59b	1.23	37.34b	1.03	
‘Cabernet Sauvignon’																
	4	26.38b	0.10	17.92b	0.69	18.70b	0.42	15.46b	0.41	13.95b	0.41	14.04b	0.60	37.66b	0.66	
	5	22.82a	0.59	15.54ab	0.47	15.79ab	0.45	13.60a	0.92	12.01ab	0.22	11.76ab	0.18	32.47ab	0.49	
	6	21.45a	1.73	14.26ab	1.12	15.22ab	1.53	12.86a	1.04	11.90ab	1.50	11.51ab	0.69	30.86ab	1.38	
	7	21.27a	1.10	14.08ab	0.27	15.15ab	0.09	12.64a	0.71	12.05ab	0.70	11.32ab	0.19	33.76ab	3.00	
	8	21.59a	0.69	14.31ab	0.46	15.36ab	0.76	12.98a	0.47	12.77ab	0.21	11.29ab	0.85	30.30ab	1.99	
	9	22.26a	0.81	15.61ab	0.23	16.80ab	0.30	14.29b	0.04	13.24b	0.60	12.66b	0.49	38.20b	0.37	
	10	22.93a	0.32	15.97ab	0.57	16.74ab	0.67	13.67ab	0.76	13.02ab	0.56	12.31b	0.51	33.47b	1.75	
	0	28.99b	1.31	19.42b	0.77	17.66b	1.06	15.86ab	0.67	14.20b	1.02	13.42b	0.38	44.01b	2.76	
	1	24.48ab	3.14	17.63ab	1.38	17.07b	0.93	15.95ab	1.01	15.15b	1.27	14.10b	0.29	47.37b	0.74	
	2	20.78a	0.96	13.45a	0.38	14.20a	0.25	12.03a	0.71	11.03a	0.62	10.49a	0.70	33.08a	2.56	
	3	21.25ab	0.46	16.20ab	0.05	15.64ab	0.83	14.10a	0.35	14.20b	0.66	13.07b	1.03	40.83ab	4.21	
‘Marselan’	4	23.53b	0.29	17.53ab	0.24	17.34b	0.20	15.49cd	0.13	15.11b	0.18	13.58b	0.46	41.75ab	3.28	
	5	16.75a	0.94	12.70a	0.50	12.91a	0.57	11.83a	0.90	11.12a	1.12	10.63a	0.70	34.17a	3.17	
	6	18.14a	1.01	13.57a	0.72	13.59a	0.67	12.78a	0.98	11.36a	0.51	10.51a	0.41	32.81a	0.99	
	7	16.22a	1.69	11.97a	1.37	11.30a	1.29	11.10a	1.22	9.47a	1.07	9.14a	0.56	28.57a	2.56	
	8	23.77ab	1.35	17.95ab	1.28	17.19b	1.28	16.96b	1.24	13.86b	2.13	13.71b	0.56	42.62ab	2.37	
	9	17.56a	0.24	12.56a	0.09	11.88a	0.50	11.62a	0.63	10.36a	0.44	9.94a	0.11	30.71a	0.20	
	10	18.40a	0.61	12.35a	0.36	13.74a	0.68	11.53a	1.48	10.94a	0.70	10.68a	1.47	33.25a	3.23	

Note: The values with different letters within each column (in each grape cultivar) are signiﬁcant difference (Duncan test, p < 0.05)

With regard to the individual oligomers, each of them presented a fluctuating decline in content in the seeds of ‘Shiraz’ and ‘Marselan’, but most of oligomers in ‘Cabernet Sauvignon’ seeds increased slightly (Table 2). Such a difference in change patterns between grape cultivars may be due to genetic regulation. As far as we know, this is the first time the changes of individual oligomeric flavan-3-ols in grape seeds are reported. In terms of the relative proportion of individual oligomer content to total flavan-3-ol monomers content at the same stage, the three cultivars showed similar profiles (data not shown). In detail, the relative proportion of dimers was around 20% and that of trimers, tetramers and pentatmers, respectively, was about 12%, and other oligomers nearly 9%. The proportion of dimers decreased faster than other oligomers. 

### 2.3. Changes in polymeric flavan-3-ols after veraison

The content of polymeric flavan-3-ols (DP > 10) in ‘Marselan’ seeds declined at harvest in comparison with that at veraison. However, in ‘Shiraz’ and ‘Cabernet Sauvignon’ seeds the polymers had no significant changes in content between these two sampling dates. It was noteworthy that flavan-3-ol polymers in the seeds of ‘Cabernet Sauvignon’ rapidly declined during the first two weeks after veraison, which was concomitant with the progressive increase of oligomer levels. Following that, the total polymer level showed little significant change, which was similar to the report of Kennedy *et al.* [23] who had found that the polymeric procyanidins in ‘Cabernet Sauvignon’ seeds containing over 30 subunits remained essentially constant during grape maturation. On the last sampling date, ‘Cabernet Sauvignon’ and ‘Marselan’ seeds contained about 230 mg/g of polymeric flavan-3-ols, much higher than that in ‘Shiraz’ grape seeds (198 mg/g). 

### 2.4. Changes in the proportions of total monomers, total oligomers, and total polymers after veraison

To better understand flavan-3-ol polymerization in maturing seeds, we assessed the relative proportion of all monomers, oligomers and polymers, respectively, to all the detected flavan-3-ols. Similar change patterns were observed in these three cultivars. The polymers accounted for the highest proportion in each cultivar, being more than 60% of total flavan-3-ols, whereas that of monomers was the lowest, about 10% (Figure 4). 

The proportion of flavan-3-ol monomers in each cultivar declined gradually during seed maturation. But those of oligomers and polymers behaved in different ways among these three grape varieties. In ‘Shiraz’ seeds, the proportion of polymers gradually increased, while that of oligomers was essentially unchanged from grape veraison to harvest. The polymers in ‘Marselan’ seeds also showed an increasing tendency in proportion during maturation, while that of oligomers presented a decreasing tendency till a week before the last sampling date, but on the last sampling date, oligomer proportion increased dramatically to its initial level. In ‘Cabernet Sauvignon’ seeds, polymers greatly declined in proportion in the first two weeks after veraison and then increased up to the initial level within the following three weeks, and after that time, this proportion remained almost constant till harvest; the relative proportion of oligomers first increased in the first two weeks after veraison and then remained around this level before harvest (Figure 4). Although differences exist between these cultivars in the change patterns of the relative proportion of these oligomers or polymers in the maturing seeds, a common tendency could be seen: the decrease in the relative proportion of flavan-3-ols with small molecular size corresponded to an increase in that of the flavan-3-ols with larger molecular size. It is thus suggested that the decrease in the relative proportion of monomers or even oligomers may contribute to the increase in that of polymers. Gaulejac *et al.* [30] have also pointed out that the increase of the proportion of polymers should be attributed to the polymerization of flavan-3-ols with lower DP. 

Several potential mechanisms of flavan-3-ol polymerization have been proposed [33]. Some studies have showed that both (-)-epicatechins and (+) catechins could be used not only as the starting units, but also as the extension units [34,35,36]. In the present study, on one hand, there existed some differences in the contents of individual monomers, but similarity in the change patterns of the relative content proportion among the detected cultivars; on the other hand, both the content of individual oligomers and the change pattern of the relative proportion varied from cultivar to cultivar, but the change pattern of the relative proportion of polymers was consistent. These results imply that the flavan-3-ol polymerization mechanism may be different, depending on grape cultivar. Up to now, it is still uncertain how to cause flavan-3-ol polymerization, by either enzyme-mediated and non-mediated reactions or both of them. Considerable further researches is needed, such as investigating more grape cultivars and screening their flavan-3-ol profiles; analyzing the mutants with specific flavan-3-ol composition so as to find defective expression of genes involved in flavan-3-ol polymerization. Besides, since flavan-3-ols with different DP characterize grape berry flavor quality, it is also interesting to compare differences and similarities in composition of flavan-3-ols with different DP between the skin and seeds in developing grape berries, as well as transcriptional regulation of genes related to biosynthesis of falvan-3-ols. The present study provides at least a technology basis for qualitative and quantitative analyses of flavan-3-ols with different DP.

## 3. Experimental

### 3.1. Chemicals and standards

The standards of C, EC, ECG and procyanidin B1 were all purchased from Sigma Chemical Co. (St. Louis, MO, USA). Methanol and acetic acid (HPLC grade) were obtained from Fisher Company (Fairlawn, NJ, USA), and deionized water (<18 MΩ resistibility) from a Milli-Q Element water purification system (Millipore, Bedford, MA, USA).

### 3.2. Preparation of samples

Grape berries of *Vitis vinifera* ‘Shiraz’, ‘Cabernet Sauvignon’ and ‘Marselan’ were collected from a single vineyard in Huailai County (40° N, 115° E), Hebei province, northwest of Beijing (China) during the 2006 growing season. The vineyard is located on the southern bank of the Guan-ting reservoir. The average annual temperature is 10.5 °C, the coldest month being January with an average temperature of −8.3 °C and the hottest month being July at an average temperature of 25 °C. There is big temperature difference between day and night. The average annual rainfall is nearly 413 mm, most of which comes in July and August. Soils in the vineyard are uniform, typified by coarse sandy loam. Row and vine spacing are 2.5 m and 1 m respectively, with the orientated in a south-north direction. The berries began coloring on Aug. 2^nd^, 3^rd^, and 6^th^ for ‘Cabernet Sauvignon’, ‘Shiraz’, and ‘Marselan’ respectively, and completed about a week later. The berries were sampled randomly at weekly intervals from the end of veraison (100% colored) to commercial harvest (24 Brix). To obtain samples representing a vineyard population, we sampled according to the method described by He *et al*. [37]. Three 100-berry samples were selected from at least seven 10-clusters at similar position of 30 whole vine selections. For this study, the seeds were separated from the rest of the grape berries. The seeds were immediately frozen in liquid nitrogen, and then ground to a fine powder with a particle size of <50 µm, freeze-dried, and stored at −80 °C until analyzed. 

### 3.3. Extraction and purification of grape seed flavan-3-ols

The extraction and purification procedures were performed as described by Gu *et al.* [21] with some modification. Briefly, the powdered sample (1.0 g) was added into a 50-mL screw-cap tube. Flavan-3-ols were extracted with mixed solvent (20 mL, acetone/water, 70:30 v/v) containing ascorbic acid (1 g/L) by shaking it followed by sonication for 10 min. Then the tube was incubated at 35 °C for 0.5 h, and centrifuged at 10,000 ×*g* for 10 min. The precipitate was extracted three times as discussed previously and the supernatants were combined. Acetone was removed by a rotary evaporation at 30 °C and the residue was dissolved in approximately 6 mL of 30% (v/v) aqueous methanol before loading onto a Sephadex LH-20 column (6 × 1.5 cm). After loading the sample, the column was washed with 30% (v/v) methanol/water (40 mL) to remove the sugars and other phenols with low molecular weight. Flavan-3-ols were obtained from the column by eluting with 70% (v/v) aqueous acetone (70 mL). The effluents were evaporated under partial vacuum at 30 ºC and the residues were freeze-dried. Then the dried residues were dissolved in methanol (HPLC grade) with a total volume of 5 mL. The methanol extract containing flavan-3-ols was filtered through 0.45 µm filters (cellulose acetate and nitrocellulose, CAN) prior to analysis by NP-HPLC-MS. For each sample, at least three independent extractions were carried out.

### 3.4. Flavan-3-ol analysis by normal phase HPLC-MS

The analysis of flavan-3-ols was carried out using an Agilent 1100 series LC and LC/MSD Trap VL mass spectrometer (Agilent Technologies, Palo Alto, CA, USA) equipped with electrospray ionization (ESI) interface, using Agilent Zorbax RX-SIL (5 μm, 2.1 × 150 mm) column protected with a Zorbax RX-SIL (5 μm, 4.6 × 12.5 mm) guard column. The mobile phase consisted of (A) methylene chloride, (B) methanol, and (C) acetic acid and water (1:1 v/v). The sample of 2 μL was injected and run at 30 °C with a flow rate of 0.2 mL/min. The gradient was 0-20 min, 14.0–25.0% B linear; 20–40 min, 25.0–33.2% B linear; 40–45 min, 33.2–86.0% B linear; 45–55 min 86.0% B isocratic; 55–60 min, 86.0–14.0% B linear followed by 10 min of re-equilibration of the column before the next run. A constant 4.0% C was kept throughout the gradient. The LC/MS conditions were at negative mode as follows: capillary voltage, 3.2 kV; cone voltage, 30 V; source temperature 150 °C; desolvation gas temperature, 300 °C. For each extraction, duplicate analyses were carried out.

EC, C, and ECG in the seeds were quantified using the respective EC, C and ECG standards as references, and oligomeric and polymeric flavan-3-ols using the standard procyanidin B1 as reference.

### 3.5. Statistical analysis

Statistical analyses were performed with the aid of SPSS 11.5 software (for Windows, license No. 30001359390). One-way analysis of variance (ANOVA) was applied to maturing seeds to verify significant changes (significant at 5% level) according to flavan-3-ols with different DP. 

## 4. Conclusions

In this study, the developmental changes in the content and the relative proportion of flavan-3-ol monomers, oligomers and polymers in seeds of ‘Shiraz’, ‘Cabernet Sauvignon’, and ‘Marselan’ grape were monitored during grape maturation. From the result we can conclude that flavan-3-ols with different molecular sizes in grape seeds behave differently during fruit ripening, and the change patterns of these compounds are cultivar-dependent, which may be genetically regulated. The genetic regulation mechanism needs to be determined in future studies. 
